# Supplementary figures and images for: Spinal Cord Injury Reduces Serum Levels of Fibroblast Growth Factor-21 and Impairs Its Signaling Pathways in Liver and Adipose Tissue in Mice
Source: Front Endocrinol (Lausanne). 2021 May 11;12:668984. doi: 10.3389/fendo.2021.668984 (PMC8147560; doi:10.3389/fendo.2021.668984)

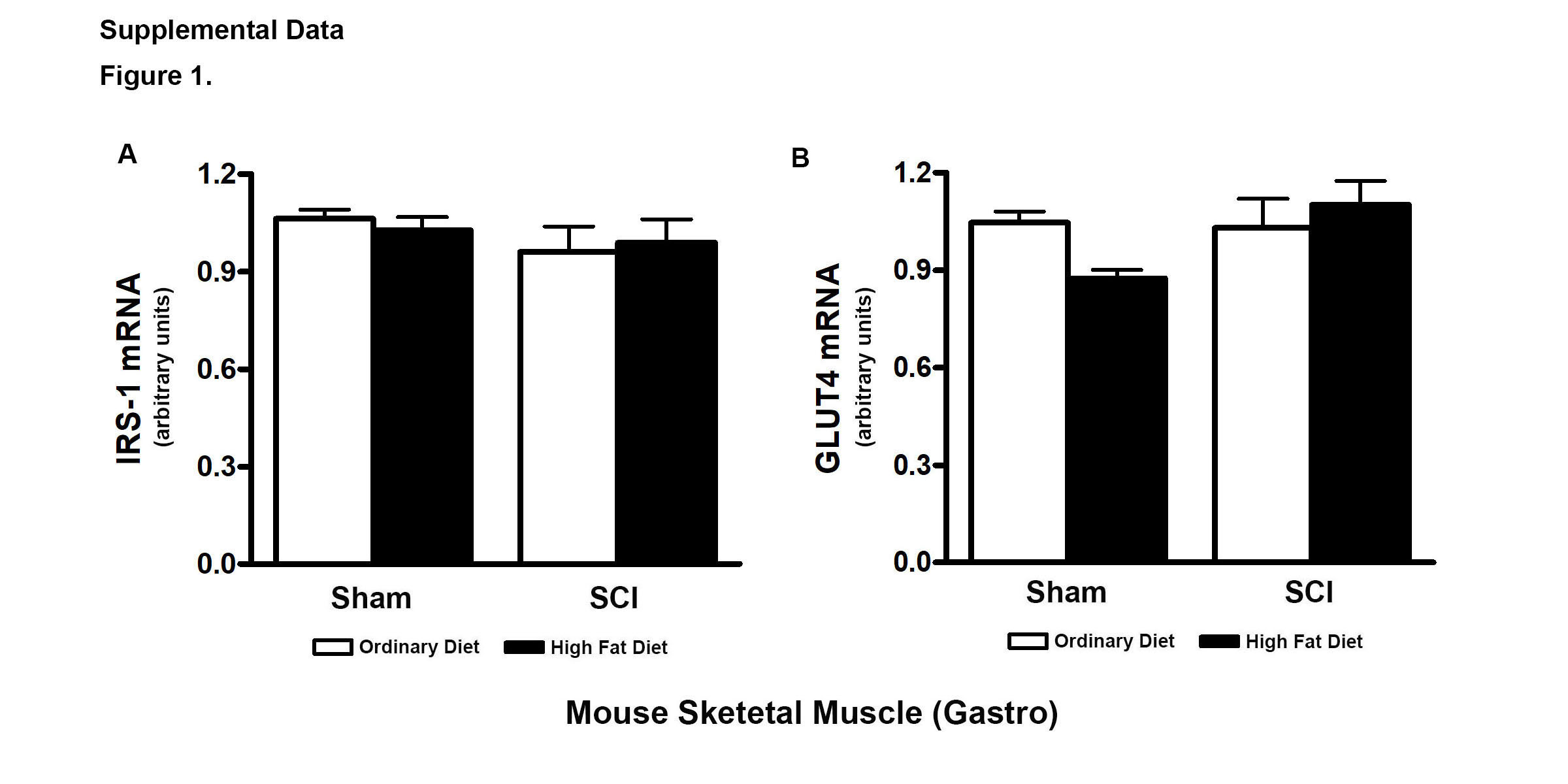

Supplement: Supplementary Figure 1 — Effect of SCI and HFD on Glut4 and IRS-1 mRNA Expression in Skeletal Muscle. Mice were fed with either ConD (White Bar) or HFD (Black Bar) after surgery. Total RNA was isolated from mouse gastrocnemius and subjected to PCR analysis. (A), IRS-1 mRNA expression; and (B), Glut4 mRNA expression. Data shown are mean values ± SEM (n = 5). [file Image_1.jpeg]

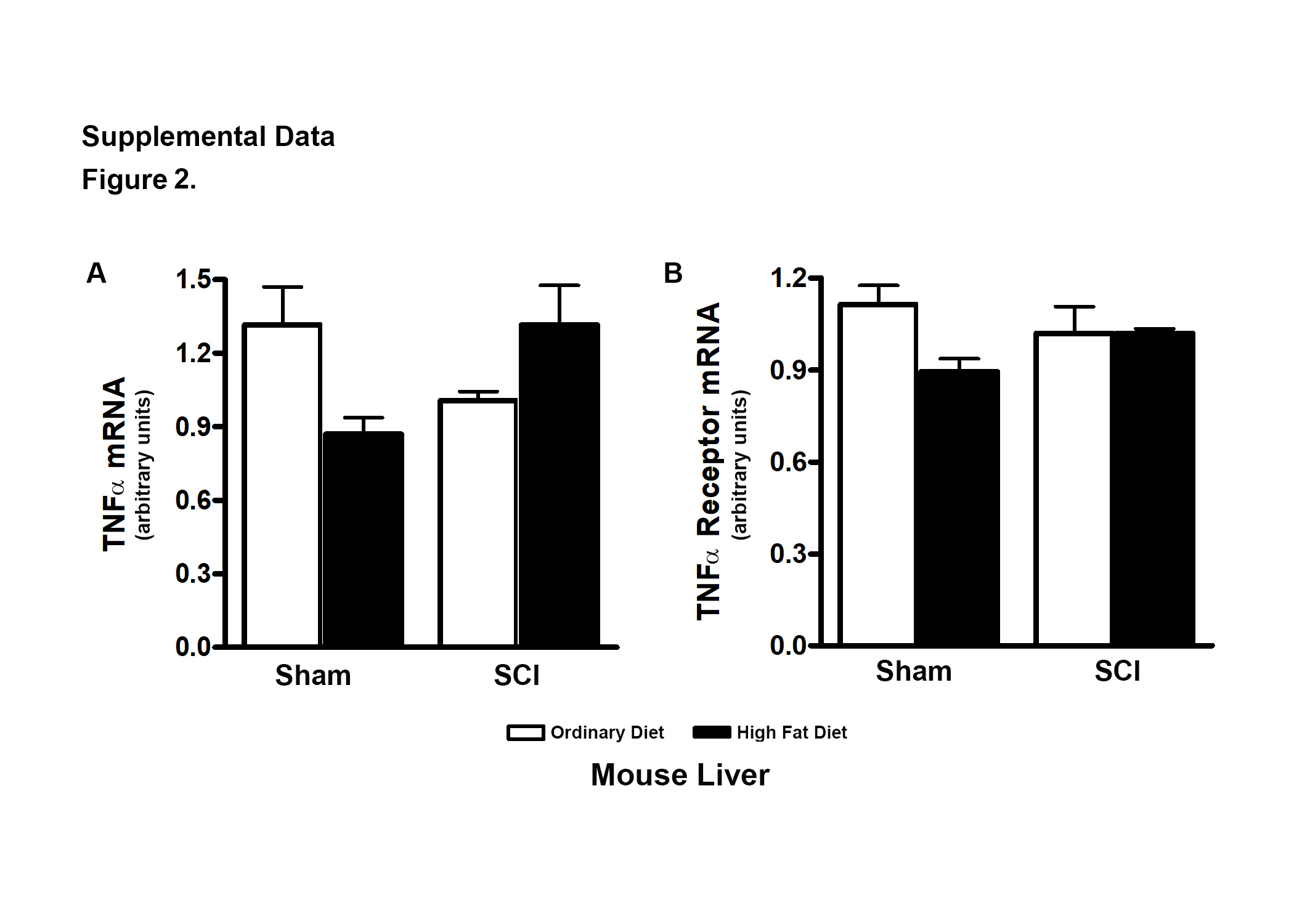

Supplement: Supplementary Figure 2 — Effect of SCI and HFD on Hepatic TNFα and TNFα receptor mRNA Expression. Mice were fed with either ConD (White Bar) or HFD (Black Bar) after surgery. Total RNA was isolated from mouse liver and subjected to PCR. (A), TNFα mRNA expression; and (B), TNFα receptor mRNA expression. Data shown are mean values ± SEM (n = 5). [file Image_2.jpeg]

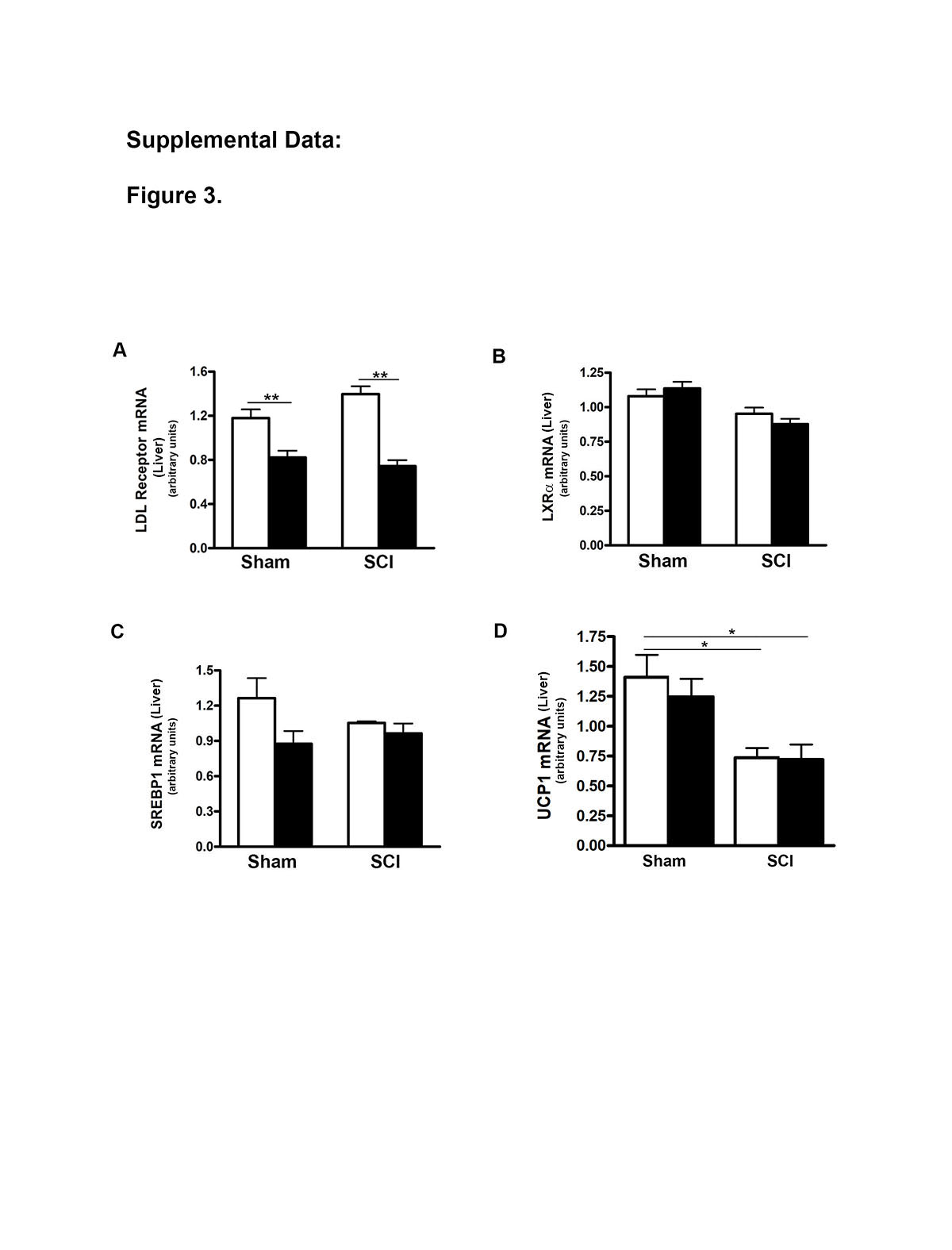

Supplement: Supplementary Figure 3 — Effects of SCI and HFD on lipid and fatty acid metabolism-related gene expression in the liver - Mice were fed with either ConD (White Bar) or HFD (Black Bar) after surgery. Total RNA was isolated from mouse liver and subjected to PCR. (A), LDL receptor mRNA; (B) , Liver X receptor-α (LXRα) mRNA; (C), SREBP1 mRNA; (D), UCP1 mRNA. Data shown are mean values ± SEM (n = 5). [file Image_3.jpeg]
