# Supplementary material for: Spinal Cord Injury Reduces Serum Levels of Fibroblast Growth Factor-21 and Impairs Its Signaling Pathways in Liver and Adipose Tissue in Mice
Source: Front Endocrinol (Lausanne). 2021 May 11;12:668984. doi: 10.3389/fendo.2021.668984 (PMC8147560; doi:10.3389/fendo.2021.668984)
Supplement: Supplementary Table 1 — Summary of Metabolic Gene Expression after SCI with or without HFD. [file Table_1.docx]

Table 1. Alterations in Metabolic Gene Expression after SCI with or without HFD

| Gene Name | Tissue Origins | SHAM-ConD | Sham-HFD | SCI-ConD | SCI-HFD |
| --- | --- | --- | --- | --- | --- |
| FGF21 Secreted Protein | Serum | 1.0 | 0.405 ** | 0.222 ** | 0.132 ** ^#^ |
| FGF21 mRNA | Liver | 1.0 | 0.219 ** | 0.403 * | 0.084 ** ^#^ |
| β-Klotho (KLB) | iFAT | 1.0 | 0.716 * | 0.362 ** | 0.173 ** ^##^ |
| β-Klotho (KLB) | oFAT | 1.0 | 3.071 * | 0.489 * | 0.305 ** |
| FGFR1 | iFAT | 1.0 | 1.195 | 0.382* | 0.411* |
| FGFR1 | oFAT | 1.0 | 2.557 | 0.324 | 0.540 ^#^ |
| Adiponectin Protein(Total) | Serum | 1.0 | 0.953 | 0.929 ** | 0.886 ** |
| Adiponectin Protein(HMW) | Serum | 1.0 | 0.628 * | 0.460 * | 0.539 * |
| Adiponectin mRNA | iFAT | 1.0 | 0.914 | 0.390 ** | 0.455 ** |
| Adiponectin mRNA | oFAT | 1.0 | 1.378 | 0.428 * | 0.168 * ^##^ |
| Leptin Secreted Protein | Serum | 1.0 | 2.950 * | 0.178 * | 0.365 |
| Leptin mRNA | iFAT | 1.0 | 2.435 * | 0.156 * | 0.097 * |
| Leptin mRNA | oFAT | 1.0 | 5.450 * | 0.562 | 0.155 * ^##^ |
| PPARγ mRNA) | iFAT | 1.0 | 0.968 | 0.475 * | 0.242 * |
| PPARγ mRNA | oFAT | 1.0 | 3.260 | 0.519 ** | 0.292 ** |
| ASC-1 mRNA | iFAT | 1.0 | 1.100 | 0.193 ** | 0.366 * |
| ASC-1 mRNA | oFAT | 1.0 | 4.290 * | 0.330 | 0.433 * |
| AdipoR2 mRNA | Liver | 1.0 | 0.768 * | 0.718 ** | 0.427 ** |
| PPARα mRNA | Liver | 1.0 | 0.876 | 0.661 | 0.739 * |
| PPARα-Total.Protein | Liver | 1.0 | 0.600 | 0.782 | 1.43 ** |
| PPARα-phos-Protein | Liver | 1.0 | 2.630 * | 3.380 ** | 2.238 ** |
| PPARα.DNA.Binding | Liver | 1.0 | 0.583 * | 0.449 ** | 0.637** |
| HMGCR mRNA | Liver | 1.0 | 0.551* | 3.010 * | 1.030 ^#^ |
| ACCα.mRNA | Liver | 1.0 | 0.145 * | 0.747 | 0.168* |
| ACCα Total Protein | Liver | 1.0 | 0.987 | 1.923 * | 1.472 |
| ACCα.phos-Protein | Liver | 1.0 | 0.291 * | 0.34* | 0.681 |
| FFA | Serum | 1.0 | 0.962 | 0.683 | 1.656 # |
| FABP4 mRNA | Liver | 1.0 | 1.011 | 1.106 | 1.844 **,^##^ |
| TNFα mRNA | iFAT | 1.0 | 1.897 | 5.820 ** | 8.032 * |
| TNFα mRNA | oFAT | 1.0 | 3.020 * | 2.54 * | 5.52 ** ^#^ |
| CD11b.mRNA | Liver | 1.0 | 1.197 | 2.401 ** | 3.787 ** |
| ALT Secreted | Serum | 1.0 | 1.556 ** | 1.733 * | 1.542 * |
| ALT mRNA | Liver | 1.0 | 1.855 * | 1.481* | 1.516 ** |
| IRS1 mRNA | iFAT | 1.0 | 1.074 | 0.542 ** | 0.403 ** |
| IRS1 mRNA | oFAT | 1.0 | 1.03 | 0.204 * | 0.208 * ^##^ |
| Glut4 mRNA | iFAT | 1.0 | 0.815 | 0.546 * | 0.293 ** |
| Glut4 mRNA | oFAT | 1.0 | 0.956 | 0.239 * | 0.164 * ^#^ |
| IRS1 mRNA | Liver | 1.0 | 0.655 | 0.421 * | 0.369 ** |
| Glycogen | Liver | 1.0 | 0.830 | 0.787 | 0.822 |
| Insulin Receptor | Gastro | 1.0 | 0.778 | 0.575 * | 0.713 * |
| Glycogen | Gastro | 1.0 | 0.694 | - 1. * | - 1. * |

* *p* < 0.05, ** *p* < 0.01 when compared to Sham-ConD; # *p* < 0.05, *p* < 0.01 when compared to SCI-ConD.
